# Rapid and cost-effective evaluation of bacterial viability using fluorescence spectroscopy

**DOI:** 10.1007/s00216-019-01848-5

**Published:** 2019-05-02

**Authors:** Fang Ou, Cushla McGoverin, Simon Swift, Frédérique Vanholsbeeck

**Affiliations:** 1 Department of Physics, The University of Auckland, Auckland, 1010 New Zealand; 2 The Dodd-Walls Centre for Photonic and Quantum Technologies, Auckland, 1010 New Zealand; 3 School of Medical Sciences, The University of Auckland, Auckland, 1023 New Zealand

**Keywords:** Bacteria, Bacterial viability, Fluorescence, Spectroscopy, Support vector regression

## Abstract

A rapid and easy method that takes advantage of an inexpensive and portable fibre-based spectroscopic system (optrode) to determine the ratio of live to dead bacteria is proposed. Mixtures of live and dead *Escherichia coli* with proportions of live:dead cells varying from 0 to 100% were stained using SYTO 9 and propidium iodide (PI) and measured using the optrode. We demonstrated several approaches to obtaining the proportions of live:dead *E. coli* in a mixture of both live and dead, from analyses of the fluorescence spectra collected by the optrode. To find a suitable technique for predicting the percentage of live bacteria in a sample, four analysis methods were assessed and compared: SYTO 9:PI fluorescence intensity ratio, an adjusted fluorescence intensity ratio, single-spectrum support vector regression (SVR) and multi-spectra SVR. Of the four analysis methods, multi-spectra SVR obtained the most reliable results and was able to predict the percentage of live bacteria in 10^8^ bacteria/mL samples between *c.* 7 and 100% live, and in 10^7^ bacteria/mL samples between *c.* 7 and 73% live. By demonstrating the use of multi-spectra SVR and the optrode to monitor *E. coli* viability, we raise points of consideration for spectroscopic analysis of SYTO 9 and PI and aim to lay the foundation for future work that uses similar methods for different bacterial species.

## Introduction

Monitoring bacterial viability is an important task in many fields of microbiological study including the monitoring of food safety and public health. Current standard assessments rely largely on the agar plate count method. A count of the viable bacteria in the sample is obtained via enumeration of the colony-forming units (CFU) following an incubation period, with the assumption that each CFU grew from one bacterium of the sample [1]. Due to the need for incubation, the agar plate count process requires 1 to 5 days [1, 2]. In addition, only the cells that can form colonies under the conditions of the experiment will be counted, thus providing no indication of dead bacteria or viable but non-culturable (VBNC) cells [1, 3, 4].

A common alternative method of analysis involves using fluorescent dyes SYTO 9 and propidium iodide (PI) which differentially stain live and dead bacteria. The signals from the dyes are typically measured using fluorescence microscopy, fluorescence-based microplate readers, or flow cytometry. Fluorescence microscopy provides direct morphological information of individual cells, but it has a small field of view; thus, the analysis of large sample volumes becomes time consuming [5, 6]. Compared to microscopy, fluorescence-based microplate readers and flow cytometry (FCM) have superior ease of use, as many of its operations can be automated and performed in parallel [7, 8]. However, the accuracy of microplate reader and FCM measurements depend on the sensitivity of the instrument and the quality of the optics, which increases with cost [9–11]. In addition, the equipment is bulky and often requires operation by trained technicians.

In pursuit of a rapid, convenient and cost-effective method for monitoring bacterial viability, the optrode has proven promising [12, 13]. The optrode is a portable fibre-based spectroscopic system that uses a stable diode pumped solid-state laser and sensitive CCD spectrometer to accurately quantify the fluorescence signals from a sample. The current (2019) setup cost of the optrode is *c.* 20K USD, which is competitive against commercial systems of fluorescence microscopy (20K–100K USD), fluorescence-based microplate readers (10K–100K USD) and flow cytometry (50K–500K USD) [13–16]. The quantitative fluorescence spectra obtained by the optrode are corrected for variations in excitation illumination and signal integration times, thus enabling the comparison of measurements from samples across a wide concentration range. The optrode measures fluorescence across the entire visible range and the sample exposure time can be varied between 8 ms and 10 s. This allows detailed characterisation of various fluorophores and how their signal changes in different experimental environments.

In this study, the optrode was used to accurately measure fluorescence emissions from bacterial samples stained with both SYTO 9 and PI. Initially to calibrate the spectral profile to percentage of live bacteria present, the ratio of the SYTO 9:PI peak intensity described in the LIVE/DEAD BacLight Bacterial Viability Kits manual [17] was used. However, our results showed that the linear relationship between the SYTO 9:PI intensity ratio and the percentage of live bacteria is not reliable and becomes variable particularly above *c.* 60% live. This observation agrees with previous studies where the SYTO 9:PI dye ratios were obtained from fluorescence-based microplate reader measurements [18–20]. We show alternative approaches to the analysis of quantitative fluorescence spectral data, to obtain with higher accuracy a measure of the percentage of live *Escherichia coli* present in a mixture of both live and dead cells.

## Materials and methods

### Bacterial culture conditions

As a model, *Escherichia coli* strain ATCC 25922 (American Type Culture Collection, Virginia, USA) was used in all experiments. *E. coli* was grown overnight then sub-cultured at 20× dilution and incubated for 1 h to reach an optical density (OD) between 0.5 and 0.6 at 600 nm (path length 1 cm), representing *c.* 4 × 10^8^ CFU/mL. All broth cultures were incubated at 37 °C in Difco tryptic soy broth (TSB; Fort Richard Laboratories, Auckland, New Zealand) and aerated with orbital shaking at 200 rpm.

### Live:dead bacterial mixtures

Live and dead bacterial mixtures were obtained using previously described methods [21]. Briefly, the sub-cultured *E. coli* in the exponential growth phase were harvested via centrifugation (4302×*g*, 10 min, 21 °C), followed by removal of supernatant and resuspension of the pellet in 3 mL of saline. Aliquots of the resuspended cells were diluted 1:9 mL in either saline for live cells, or 70% isopropanol for dead cells. Each bacterial suspension was shaken at 200 rpm at 28 °C for 1 h. The live and dead bacterial cells were harvested via centrifugation (4302×*g* 10 min, 21 °C), followed by removal of the supernatant and resuspension of the pelleted cells in 20 mL of saline. After three washing cycles, each of the live and dead cell suspensions was diluted to achieve a concentration of *c.* 1 ×10^8^ bacteria/mL; equivalent to diluting the sample to a final OD of 0.168 ± 0.063 at 600 nm. These live and dead bacterial suspensions were used either directly or diluted to 1 × 10^7^ bacteria/mL, to prepare mixtures with live:dead proportions corresponding to 0, 2.5, 5, 10, 25, 50, 75, 100% live bacteria.

### Fluorescent dye staining and treatment of unbound dyes

BacLight LIVE/DEAD Bacterial Viability and Counting Kits (Invitrogen, Molecular Probes, Carlsbad, CA, USA; L34856) were used in all experiments. The kit comprises a vial of microsphere suspension, and two nucleic acid dyes SYTO 9 and propidium iodide (PI) that label live and dead bacteria, respectively. For each experiment, saline was used as diluent to make working solutions of SYTO 9 and PI with concentrations of 33.4 μM and 0.4 mM, respectively. The microsphere suspension was sonicated (SC-120 sonicator, Sonicor, NY, USA) for 10 min in a water bath and gently vortexed at 500 rpm prior to use. For each sample, 50 μL each of the SYTO 9 and PI working solutions, and 10 μL of the microsphere suspension, were aliquoted into an empty microcentrifuge tube. Subsequently, 900 μL of each bacterial sample was added to each tube then gently vortexed at 500 rpm in the dark for 15 min at room temperature, to allow dye-bacteria binding.

### Flow cytometry protocols

Reference measurements of all samples were obtained by the LSR II Flow Cytometer (BD Biosciences, San Jose, CA, USA), using a method we devised and for which we evaluated its sensitivity and accuracy against plate counting [21]. Plate counting is not accurate enough as a reference method for the current study. In brief, samples were excited with a solid-state Sapphire 488-nm blue laser with 20 mW power (Coherent, CA, USA). Standard dichroic filters were used to direct light to the appropriate detectors in the flow cytometer. SYTO 9 fluorescence was collected using a 505-nm longpass filter and a 530/30-nm bandpass filter. PI fluorescence was collected using a 685-nm longpass filter and a 695/40 bandpass filter. The threshold was set to side scatter at 200 and the bead count per second was plotted to monitor for any disturbance or blockages. The measurement duration was 150 s and the flow rate was set to *c.* 6 μL/min. Gating was done in the red fluorescence vs green fluorescence dot plot to obtain the percentage of live and dead bacteria in each sample.

### Optrode protocols

The fluorescence dye emission spectra of bacterial samples were quantified using a fibre-based spectroscopic system called the optrode [12, 13]. Excitation is achieved by a 473-nm solid-state laser with *c*. 10 mW power. Signal variation caused by photobleaching is minimised by synchronisation of the laser shutter with the spectrometer using a data acquisition (DAQ) card. Using a 2 × 2 fibre coupler, the laser irradiates both the sample and a photodiode that monitors the laser power fluctuations. Fluorescence excitation and detection are achieved by a single fibre probe (200-μm diameter, 0.22 NA; Thorlabs Inc., Newton, NJ, USA). A 495-nm longpass filter removes the excitation line before signals reach the Ocean Optics QE65000 CCD spectrometer.

### Spectra acquisition and pre-processing

*N = 56* standard bacterial samples (*n =* 159 optrode measurements) and *N =* 27 test set samples (*n =* 80 optrode measurements) were analysed, on average each sample was measured three times. The duration of each optrode measurement was 10 s and each consisted of 500 spectra collected consecutively using a 20 ms integration time per spectrum; these optrode measurements allowed photobleaching behaviour to be observed. The instrument dark noise was removed, and all spectra were normalised to 8 ms integration time and 10 mW laser power. The averaged background spectrum obtained from saline was subtracted from each sample spectrum. Occasionally, anomalous optrode measurements, with noticeably higher or lower intensity compared to the rest of the optrode measurements collected from the sample, were obtained. The occurrences of anomalous optrode measurements were substantially reduced by thorough cleaning of the probe. The anomalous optrode measurements were excluded from analysis, noted, and are not included in the optrode measurement count (*n*) stated in the beginning of this section. The remaining fluorescence spectra were subsequently mean-centred with respect to the average of the appropriate training set spectrum, and from each optrode measurement, up to 5 spectra were chosen from the series of 500 and used in algorithms to predict the percentage of live and dead bacteria measured by FCM.

### Fluorescence photobleaching rates

Fluorescence photobleaching rates were obtained from dual-stained bacterial samples at concentration of *c.* 10^8^ bacteria/mL. In the dual-stained dead bacterial samples, while the PI will be mostly bound (mB) and SYTO 9 will be mostly unbound (mU), it is likely that some PI will remain unbound and some SYTO 9 will be bound. On the other hand, it is expected that in the dual-stained live bacterial samples, some SYTO 9 will remain unbound. The mB-SYTO 9 and mU-PI photobleaching half-lives were obtained from fluorescence emission of dual-stained live bacteria. Vice versa, the photobleaching half-lives of mU-SYTO 9 and mB-PI were obtained from fluorescence emission of dual-stained dead bacteria. The fluorescence intensity of the dyes over a 10-s duration were measured using the optrode and modelled by a double exponential to find the photobleaching half-lives of the dyes in each situation (*N = 6* bacterial samples, *n = 17* optrode measurements, on average three per sample). The *t* test was performed in Python to evaluate whether the photobleaching half-life of the dyes in bound or unbound states were significantly different.

### Data analysis

To find a suitable technique for predicting the percentage of live bacteria in a sample, four analysis methods were assessed and compared: SYTO 9:PI intensity ratio, adjusted dye ratio [22], single-spectrum support vector regression (SVR) and multi-spectra SVR. The four methods were evaluated by external validation using test set samples. The *R*^2^, standard error, root mean square error (RMSE) and explained variance were found.

### Dye ratio

In this work, the ‘dye ratio’ refers to the SYTO 9:PI integrated intensity ratio described in the LIVE/DEAD BacLight Bacterial Viability Kits manual [17], shown on the right-hand side of Eq. (1):

1 $$ \%\mathrm{live}\propto \frac{\mathrm{SYTO}\ 9}{\mathrm{PI}} $$

where *%live* corresponds to the FCM-measured percentage of live bacteria in the sample, and *SYTO 9* and *PI* represents the integrated intensity of SYTO 9 and PI, respectively. The regions of intensity integration corresponded to the fluorescence peak of the dyes, which were between 509–529 nm for SYTO 9 and 609–629 nm for PI. Mathematically, *%live* is equivalent to the number of live cells divided by the total number of cells, which is not reflected by *SYTO 9/PI*. The reason for this becomes clear when assuming the ideal behaviour of the dyes where the live and dead bacteria will be mostly labelled by SYTO 9 and PI, respectively [17]. If PI is expected to only stain dead cells, then the denominator of Eq. (1) does not accurately represent the signal from the total amount of live and dead bacteria present in the sample. Nonetheless, according to the user manual [17], the dye ratio was expected to yield a linear relationship with the percentage of live bacteria in the samples.

### Adjusted dye ratio

The adjusted dye ratio is built upon the dye ratio in Eq. (1). Most of the PI signal from bacterial samples is expected to be from dead cells, whose proportion can be represented by *%dead* or ‘100 − *% live*’:

2 $$ \frac{\%\mathrm{live}}{100-\%\mathrm{live}}\propto \frac{\mathrm{SYTO}\ 9}{\mathrm{PI}} $$

The above expression, Eq. (2) can be rearranged to:

3 $$ \%\mathrm{live}\propto \frac{100\times \mathrm{SYTO}\ 9/\mathrm{PI}}{1+\mathrm{SYTO}\ 9/\mathrm{PI}} $$

The right-hand side of Eq. (3) is referred to as the *adjusted dye ratio*, and this value is calculated and compared to the percentage of live cells present in each sample. It is worth noting that in cases where there is a low percentage of live bacteria present, the signal of SYTO 9 relative to PI will be small and the denominator of Eq. (3) will tend towards 1. Thus, in situations where the percentage of live bacteria is low, the adjusted dye ratio can be approximated to the dye ratio in Eq. (1). An extensive comparison of experimental results obtained using the dye ratio and the adjusted dye ratio has been made [23].

### Single-spectrum SVR

The first spectrum from each optrode measurement was used as input for the SVR algorithm, which maps the input data onto a high-dimensional feature space, then constructs a regression model in the feature space [24]. Unlike least squares regression which aims to only minimise the observed training error, SVR seeks to minimise both the observed training error and a regularisation value that controls the complexity of the model [25]. The ε-SVR from Python’s Scikit-learn library [26] was applied using the linear kernel. The generalisation ability of ε-SVR is controlled by two parameters ε and C, which defines the margin of tolerance and the penalty factor, respectively.

Grid search and group K-fold cross-validation (GKCV) were implemented to find the best estimator values for the ε and C parameters that minimised the mean squared error of the predictions. Grid search was applied to search over various parameter values of both ε and C to find the estimators that minimised the mean squared error of the predictions. This process was optimised by GKCV, where the spectral training dataset was split into groups according to the *M* experiments performed to collect the data at each bacterial concentration, which were 3 and 4 for the 10^7^ and 10^8^ bacteria/mL samples, respectively. Then, *M* iterations of GKCV were performed in each iteration. One group was held out as the internal test set while the remaining were used as the training set. The final combination of ε and C values that minimised the RMSE of the predictions was chosen.

### Multi-spectra SVR

Various combinations of 2, 3 or 5 spectra obtained at different time points in one optrode measurement were concatenated and analysed by SVR. Five timepoints were investigated that corresponded to: the first 20 ms following fluorescence excitation, and the photobleaching half-lives of mB-SYTO 9, mB-PI, mU-SYTO 9 and mU-PI. To find the spectral combination which returned the lowest RMSE, initial assessments by GKCV were completed using default ε and C values (of 0.1 and 1, respectively). Subsequently, the ε and C parameters of the SVR model that used the chosen spectral combination were optimised, using grid search and GKCV.

#### Data availability

The datasets generated during and/or analysed during the current study are available from the corresponding author on reasonable request.

## Results

### Fluorescence spectra and the SYTO 9 and PI peaks

**Fig. 1:**
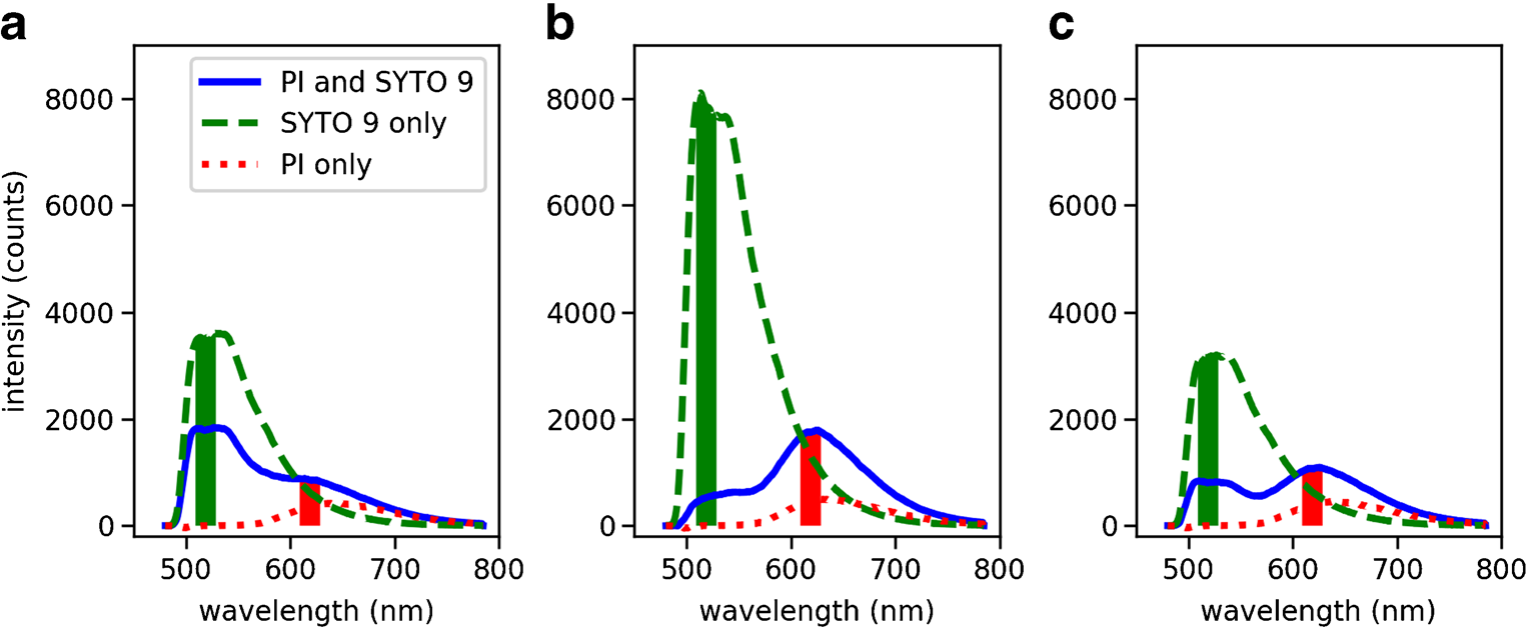
Exemplar spectra showing the difference in spectral profile obtained from samples containing 10^8^ bacteria/mL of live (**a**), dead (**b**) and 50:50 live and dead (**c**) bacteria. The bacterial samples were stained using PI only, SYTO 9 only or both PI and SYTO 9. The shaded regions near 510 nm and 610 nm represent the intervals used to obtain the integrated intensity of SYTO 9 and PI, respectively

The SYTO 9 and PI emissions of bacterial samples vary as the relative concentration of live and dead bacteria in the sample changes. Figure 1 shows spectra obtained from *E. coli* samples at concentration of 10^8^ bacteria/mL, stained using either SYTO 9 alone, PI alone, or both SYTO 9 and PI. The fluorescence peaks of SYTO 9 and PI are observed between 509–529 nm and 609–629 nm, respectively. Comparing the spectra of samples stained using SYTO 9 only: the SYTO 9 peak intensity more than doubles from the 100% live sample to the 100% dead sample. On the other hand, there is a slight peak intensity decrease of *c.* 13% from the 100% live sample to the 50% live sample. Samples stained using only PI showed lower peak intensity than those stained using only SYTO 9; however, the PI fluorescence is enhanced in the presence of SYTO 9.

### Fluorescence photobleaching rates

To obtain the photobleaching half-lives of SYTO 9 and PI, dual-stained live or dead bacterial suspensions at concentration of 10^8^ bacteria/mL were measured using the optrode. The mean photobleaching half-lives of SYTO 9 compared to PI, and in their mostly bound (mB) versus mostly unbound (mU) states, were not significantly different (*p* values > 0.05). Nonetheless, the photobleaching half-lives provide an indication of time points to inspect for spectral changes that occur over time, which is useful in the multi-spectra SVR analysis. The average photobleaching half-life of mB- or mU-SYTO 9 and PI are summarised in Table 1.

**Table 1:** The average photobleaching half-lives of SYTO 9 and PI ± the standard error, obtained from dual-stained live or dead bacterial samples with concentration of 10^8^ bacteria/mL

State of dye	Photobleaching half-life (ms)	
	SYTO 9	PI
Mostly bound (mB)	1244 ± 97	1124 ± 115
Mostly unbound (mU)	1472 ± 165	1084 ± 29

The photobleaching half-lives for mostly bound-SYTO 9 and mostly unbound-PI were obtained from dual-stained live bacteria, whereas those of mostly unbound SYTO 9 and mostly bound-PI were obtained from dual-stained dead bacteria

### Regression models: dye ratio, adjusted dye ratio, single-spectrum SVR and multi-spectra SVR

The spectral training data for 10^8^ bacteria/mL samples (*N* = 32 samples; *n* = 91 optrode measurements, on average three per sample) were obtained from four experiments. The spectral training data for 10^7^ bacteria/mL samples (*N* = 24 samples; *n* = 68 measurements, on average three per sample) were obtained from three experiments. To establish a method for correlating the spectral changes to variations in proportions of live:dead bacteria, four regression models were compared which used: the dye ratio, adjusted dye ratio, single-spectrum SVR and multi-spectra SVR.

**Fig. 2:**
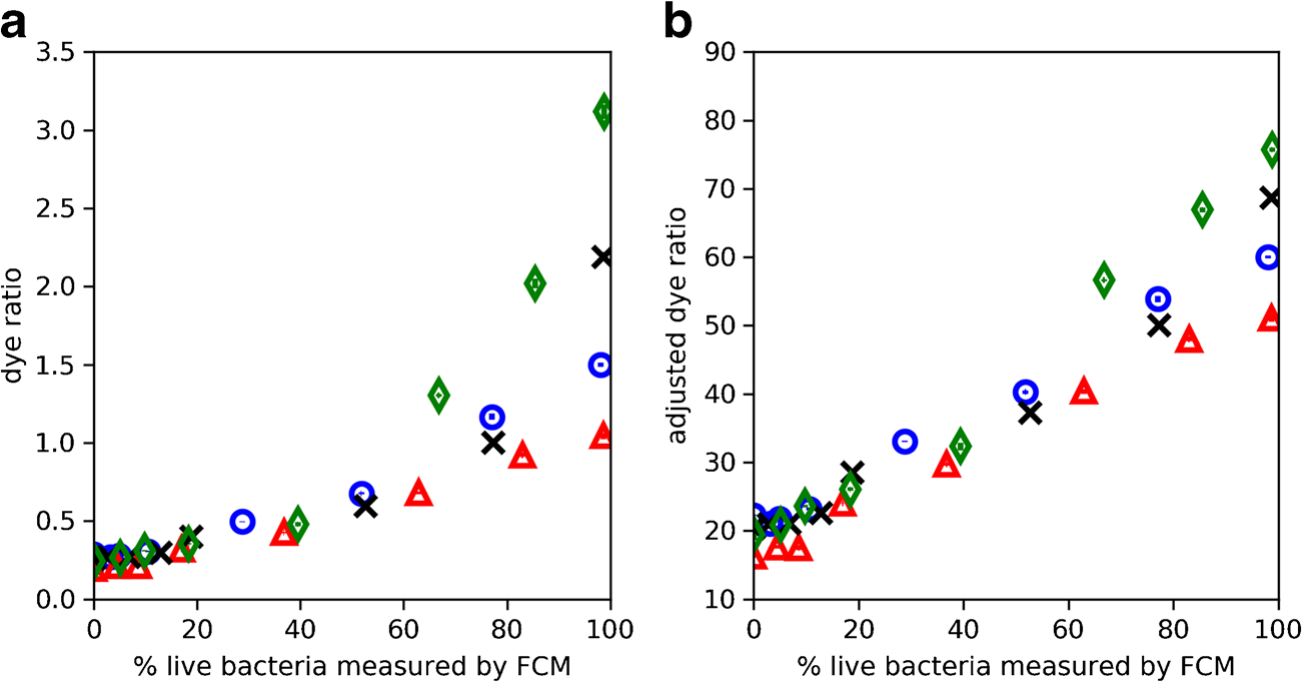
The dye ratio (**a**) and adjusted dye ratio (**b**) as a function of the percentage of live bacteria in the 10^8^ bacteria/mL training samples. Four types of markers represent the separate experiments from which the data were collected

Figure 2 shows the dye ratio and adjusted dye ratio as a function of the percentage of live bacteria in 10^8^ bacteria/mL training samples. Overall, at 10^8^ bacteria/mL, the adjusted dye ratio has a much more linear relationship with percentage of live bacteria, compared to the dye ratio. The relationship between the dye ratio and percentage of live bacteria appears nonlinear beyond *c.* 60% live. On the other hand, the adjusted dye ratio maintains a roughly linear relationship with the percentage of live bacteria, but the variations in results are greater beyond 60%. The *R*^2^ were − 1.1 and 0.67 for the dye ratio and adjusted dye ratio models, respectively, with the negative *R*^2^ indicating that the fit is worse than the mean of the data [26]. Analysis using the dye ratio and adjusted dye ratio were not useful for 10^7^ bacteria/mL samples as the results were highly variable.

Fluorescence spectra obtained at various time points corresponding to the photobleaching half-lives of the dyes in mB or mU states were concatenated with the spectrum recorded in the first 20 ms (S_1_) and analysed in multi-spectra SVR (Table 2). For the analysis of 10^8^ bacteria/mL samples, a combination of S_1_ and the spectrum taken at the photobleaching half-live of mB-SYTO 9 (1244 ms) returned the lowest error value in GKCV. Single-spectrum SVR analysis of 10^7^ bacteria/mL samples returned the lowest RMSE for 10^7^ bacteria/mL samples, slightly lower than using a combination of S_1_ and the spectrum taken at the photobleaching half-life of mB-PI (1124 ms), which returned the lowest error value of multi-spectra SVR.

**Table 2:** Combinations of spectra used in multi-spectra SVR analysis

Number of spectra used	Time points used	Pre-optimised RMSE results for 10^8^ bacteria/mL samples	Pre-optimised RMSE results for 10^7^ bacteria/mL samples
1	S_1_ (single-spectrum SVR)	20.0	21.6
2	S_1_, mB-SYTO 9 T_1/2_	19.85	23.55
	S_1_, mB-PI T_1/2_	20.73	22.87
	S_1_, mU-SYTO 9 T_1/2_	20.67	23.29
	S_1_, mU-PI T_1/2_	20.61	22.92
3	S_1_, mB-SYTO 9 T_1/2_, mB-PI T_1/2_	20.70	23.32
	S_1_, mU-SYTO 9 T_1/2_, mU-PI T_1/2_	22.07	22.96
5	S_1_, mB-SYTO 9 T_1/2_, mB-PI T_1/2_, mU-SYTO 9 T_1/2_, mU-PI T_1/2_	21.94	23.29

S_1_ represents the spectrum recorded in the first 20 ms of the measurement, and T_1/2_ represents the measured photobleaching half-life of the associated fluorophore in its mostly bound (mB) or mostly unbound (mU) state. The RMSE values of 10^8^ and 10^7^ bacteria/mL samples were obtained via GKCV prior to parameter optimisation of the SVR algorithm

The single-spectrum SVR and multi-spectra SVR regression models both obtained linear relationships for modelling the percentage of live bacteria, when using 10^8^ or 10^7^ bacteria/mL training samples. Grid search and GKCV returned 0.5 and 0.001 as the optimal values for SVR hyper parameters C and ε, respectively. The RMSE and explained variance of the predictions are summarised in Table 3.

**Table 3:** The RMSE and explained variance of the single-spectrum SVR and multi-spectra SVR models in modelling the percentage of live bacteria in training samples

Property modelled	Regression models	10^8^ bacteria/mL samples		10^7^ bacteria/mL samples	
		RMSE	Explained variance	RMSE	Explained variance
% live	Single-spectrum SVR	19.95	0.85	21.56	0.91
	Multi-spectra SVR	19.79	0.87	22.86	0.91

Multi-spectra SVR model for the analysis of 10^8^ bacteria/mL samples used a combination of the spectrum recorded in the first 20 ms (S_1_) and the spectrum taken at the photobleaching half-live of mostly bound-SYTO 9 (1244 ms); multi-spectra SVR model for the analysis of 10^7^ bacteria/mL samples used a combination of the spectrum taken at S_1_ and the spectrum taken at the photobleaching half-life of mostly bound-PI (1124 ms)

### Validation of SVR models using test set samples

The single-spectrum and multi-spectra SVR models were validated and compared using external test set samples (*N* = 27 samples; *n* = 80 optrode measurements, on average three per sample) collected from two blind experiments. The predictions obtained from multiple spectral measurements of each sample were averaged then compared to the percentage of live bacteria measured by FCM. Both the single-spectrum and multi-spectra SVR models predicted the percentage of live bacteria in the samples reasonably well. Results of the comparison between the two SVR models are shown in Table 4 and overall, the predictions obtained using multi-spectra SVR deviated less from the expected values compared to those obtained using single-spectrum SVR. Both SVR models performed better in predicting the percentage of live bacteria in samples with concentration of 10^8^ than 10^7^ bacteria/mL. It was observed that the percentage live predictions for 10^7^ bacteria/mL samples containing less than *c.* 7% or more than 73% returned percentage values that were negative or above 100%. These were considered as invalid predictions and excluded.

**Table 4:** Evaluation of the single-spectrum and multi-spectra SVR models in predicting the percentage of live bacteria in 10^8^ and 10^7^ bacteria/mL samples. Invalid predictions refer to model outputs that were negative or above 100%

SVR model	Time points used in SVR model	Concentration of samples (bacteria/mL)	Standard error	% of samples with predictions within 2 SE	No. of samples with invalid predictions
Single-spectrum	S_1_	10^8^	5.5	100	0
	S_1_	10^7^	11.7	100	3
Multi-spectra	S_1_, mB-SYTO 9 T_1/2_	10^8^	4.7	91	0
	S_1_, mB-PI T_1/2_	10^7^	8.2	100	3

**Fig. 3:**
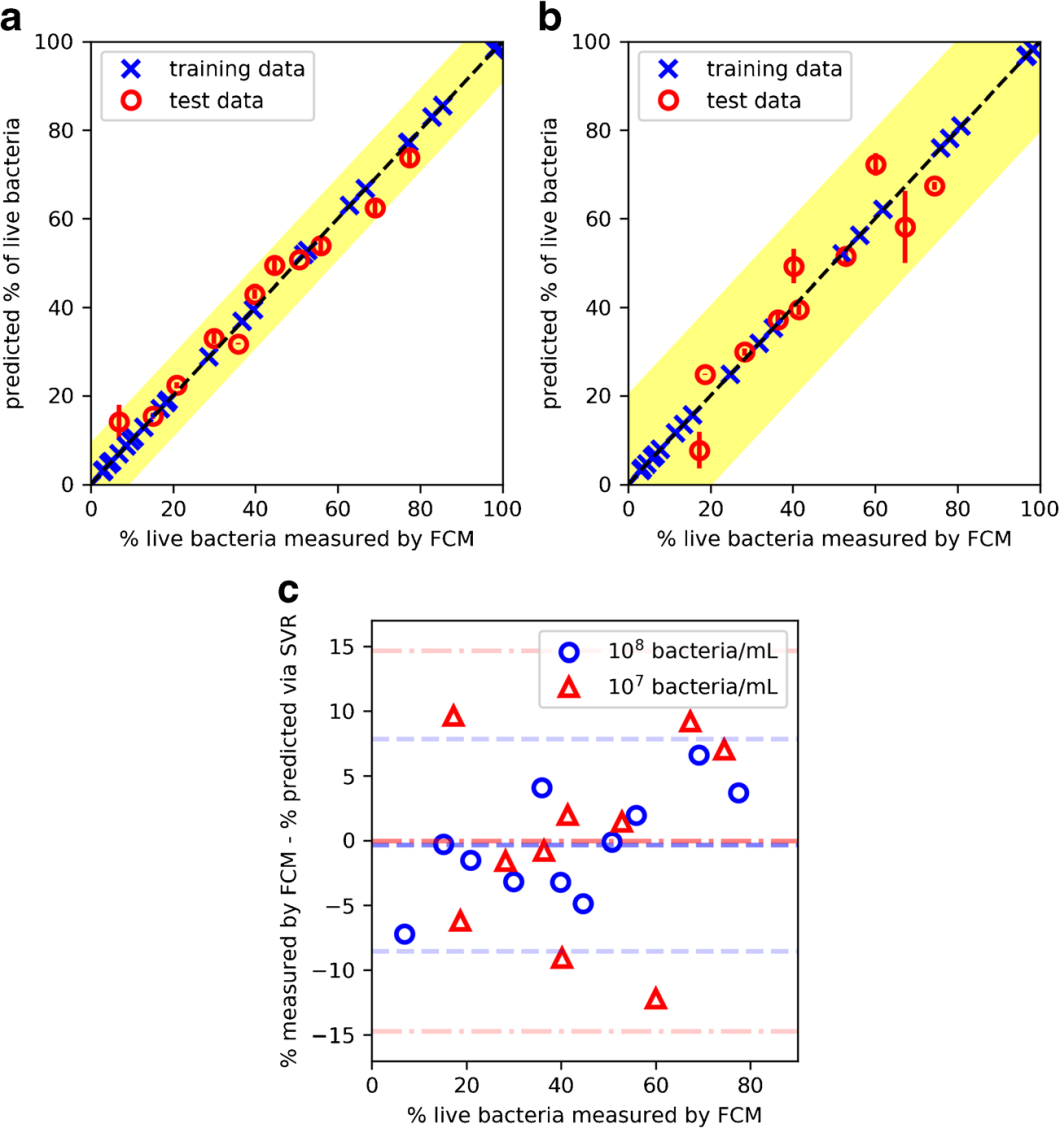
Evaluation of the multi-spectra SVR analysis method and comparison to the reference flow cytometry method. The percentage of live bacteria predicted using the multi-spectra SVR model compared to that measured by FCM is shown in parts (a) and (b). The samples contain a mixture of live and dead *E. coli* cells, with concentration of (a) 10^8^ or (b) 10^7^ bacteria/mL. The dashed line in parts (a) and (b) marks the 1:1 relationship between the predicted percent live and that measured using FCM. The shaded area represents the region of plus or minus two standard errors of the 1:1 line. The standard error in replicate measurements is represented by the vertical and horizontal error bars. A few 10^7^ bacteria/mL samples returned invalid predictions that were negative or above 100%, which were excluded. Part (c) plots the differences between the reference flow cytometry method and the multi-spectra SVR analysis method. The bias of − 0.35% and − 0.07% for 10^8^ and 10^7^ bacteria/mL samples, respectively, is represented by the horizontal dashed lines near the *X*-axis. The 95% confidence intervals for 10^8^ bacteria/mL samples are shown by the horizontal dashed lines at − 8.5% and 7.8%, and those for 10^7^ bacteria/mL samples are shown by the lines at − 14.8% and 14.6%

Predictions of the percentage of live bacteria in test set samples using multi-spectra SVR are shown in Fig. 3 (a) and (b). All predictions lie within the 95% confidence interval of each model, or ±2 standard errors of the 1:1 line [27], except for one prediction of a 10^8^ bacteria/mL sample containing low percentage of live cells that was below 7%. Figure 3 (c) shows the Bland-Altman plot of the differences between the reference FCM method and the multi-spectra SVR analysis method, as a function of the FCM measurements. The analysis showed that there is no major bias, as the mean difference between the two methods were both close to zero, − 0.35% and − 0.07% for 10^8^ and 10^7^ bacteria/mL samples, respectively. It is expected that 95% of differences will be between − 8.5 and 7.8% for 10^8^ bacteria/mL samples, and − 14.8 and 14.6% for 10^7^ bacteria/mL samples. Normality of the differences was verified using the Shapiro-Wilk test.

## Discussion

In this study, we showed several approaches to obtaining the ratio of live:dead *E. coli* in a mixture of both live and dead, from analyses of its fluorescence spectra. Spectral measurements were obtained using a portable and cost-effective lab-built optrode system that accurately quantifies fluorescence signals in near real time. Fluorescence was measured from SYTO 9- and PI-stained *E. coli* samples with varying proportions of live and dead cells, and the percentage of live cells present was predicted. To determine a suitable method to perform the predictions, four analysis methods were investigated: SYTO 9:PI intensity ratio, adjusted dye ratio, single-spectrum SVR and multi-spectra SVR. We examined the applicability and performance of these analysis methods and propose a general optrode protocol for measuring percentage of live bacteria using multi-spectra SVR.

### Fluorescence profiles of SYTO 9 and PI

Both SYTO 9 and PI were used to stain the bacterial samples in this study; however, the fluorescence profiles of samples stained using only SYTO 9 or PI were also examined (Fig. 1). The significantly higher SYTO 9 emissions in the presence of dead compared to live *E. coli* demonstrate the necessity of a counter-dye such as PI to enable quantitative fluorescence analysis of *E. coli* cells. Counterstaining may be required for most Gram-negative bacteria as the effect of stronger SYTO 9 staining of dead Gram-negative cells has also been reported in *Pseudomonas aeruginosa* [18]. It has been suggested that the strong SYTO 9 emission in dead cells could be due to its difficulty in crossing the outer membranes of Gram-negative bacteria [28], or that it is actively exported out of the cell [18]. Another possible explanation is that as these cells die, their nucleic acids adopt an open structure that is more accessible to SYTO 9 binding [29].

In the presence of PI, the SYTO 9 intensity in the dead *E. coli* samples decreased while the PI signal increased, compared to staining with the dyes individually, thus enabling the distinction between live and dead *E. coli* and subsequent quantification. Between the samples containing different composition of live and dead bacteria, there are some noticeable changes in the shape of the SYTO 9 peak emission. The SYTO 9 emission from the counter-stained 100% and 50% live samples show a subtle dual-peak at 498/501 nm, corresponding to the emission of the dye bound to DNA/RNA [30]. However, the dual-peak profile of SYTO 9 is not noticeable in the 100% dead sample. By accurately measuring the emission spectrum of counter-stained bacterial samples with the optrode, it is possible to obtain information about its composition from the dye intensities as well as the subtler changes in spectral shape. Initially, the dye ratio and adjusted dye ratio analysis were used to test the performance of simple methods that are primarily based on using the information about the peak dye intensities. Subsequently, to consider the information in peak dye intensities and the small changes in spectral shape, SVR was applied. SVR is a multivariate method that use information from the entire spectral window and thus can better characterise the spectral changes in relation to changes in the composition of the samples.

When two fluorescent dyes with different photobleaching half-lives are used together, prolonged exposure will alter the ratio of the two emission peaks and bias the result. Experiments were conducted to examine the photobleaching rates of SYTO 9 and PI in different bacterial samples and establish the effect of photobleaching on the SYTO 9:PI fluorescence ratio. The photobleaching half-lives of SYTO 9 and PI were both found to be > 1 s in their mB or mU states (Table 1). Thus, the effects of photobleaching on the spectrum measured in the first 20 ms are negligible. In addition, our results showed that the photobleaching half-lives of SYTO 9 compared to PI, and in the bound versus unbound states, were not significantly different. While the photobleaching half-lives themselves may not provide sufficient information for distinguishing between SYTO 9 and PI or their binding states, they nonetheless represent useful time points for investigating spectral shape changes. As the bacterial composition of samples change, the ratio of bound to unbound SYTO 9 and PI also changes, and this will influence the overall photobleaching rate of the samples. Thus, the extent of how much the signal has photobleached can provide additional clues about the proportions of live or dead bacteria present in the samples.

### Dye ratio and adjusted dye ratio

The dye ratio analysis is based on the method proposed in the product information guide of the LIVE/DEAD BacLight Bacterial Viability Kits [17]. However, as shown in Fig. 2(a), the relationship between the dye ratio and percentage of live bacteria only appears linear in a narrow range where samples contained low percentage of live cells. This is expected, considering the ideal behaviour of the dyes where SYTO 9 and PI stains live and dead cells, respectively. The percentage of live bacteria is equivalent to the number of live cells divided by the total cell number. In the dye ratio (Eq. 1), the SYTO 9 intensity in the numerator is related to the abundance of live cells however, the PI intensity in the denominator does not represent the total cell number as PI should only stain dead cells. Following this observation, the adjusted dye ratio was derived (Eq. 3), and its results are shown in Fig. 2b. Although the relationship remains somewhat linear between the adjusted dye ratio and the percentage of live bacteria, the variations in results increase above *c.* 60% live. One reason for why the adjusted dye ratio is not linear across the entire range is due to a breakdown of the assumption that there is no interaction between the two dyes, e.g. fluorescence from SYTO 9 can excite PI [2]. Nonetheless, the adjusted dye ratio may be useful as a simple method for providing a rough indication of the proportion of live bacteria present in 10^8^ bacteria/mL bacterial samples.

### Single-spectrum and multi-spectra SVR

Multi-spectra SVR performed slightly better in modelling the training samples and evaluating the test set samples, as they returned lower standard error values than the single-spectrum SVR. Compared to single-spectrum SVR, more time information is included in the input to the multi-spectra SVR models which may be valuable to improving the predictive power of the SVR algorithm. The additional spectra used in multi-spectra SVR were chosen from measurements taken at times corresponding to the photobleaching half-life of the dyes in their bound or unbound states (Table 1), which provide time points for inspecting changes in spectral shape.

To find the spectral sequence for multi-spectra SVR that gives the lowest RMSE, several combinations of 2, 3 or 5 spectra obtained at different time points in the optrode measurement were concatenated and used as input to the SVR algorithm. The spectrum measured in the first 20 ms was included in all multi-spectra SVR analyses, as the difference in its signal intensity is mostly due to dye/bacteria interactions with minimal effects of photobleaching. In a few circumstances, the use of 2-spectra as the input obtained results with RMSE smaller than or close to that of the single-spectrum SVR. However, the use of 3 or 5 concatenated spectra obtained worse results than single-spectrum SVR (Table 2). An explanation for this is that the spectra obtained at subsequent additional time points do not add further compositional information and rather just introduce added spectral noise. For the multi-spectra SVR to be valuable, it requires a choice of time points for spectral input that is a balance between introducing new useful information and not adding redundant data.

The chosen input for multi-spectra analysis of 10^8^ bacteria/mL samples was a combination of the first spectrum and the spectrum recorded at the photobleaching half-live of mB-SYTO 9 (1244 ms). In these samples, the SYTO 9 peak intensity varies greatly according to the proportion of live bacteria present, and the spectrum taken at 1244 ms provides additional time information of this signal. On the other hand, the first spectrum and the spectrum recorded at the photobleaching half-life of mB-PI (1124 ms) were chosen for the multi-spectra analysis of 10^7^ bacteria/mL samples. There was little obvious change in the SYTO 9 or PI peak intensity in the 10^7^ bacteria/mL samples as its proportion of live:dead cells changed. However, in the 10^7^ bacteria/mL samples, the intensity of the PI peak was observed to dominate that of the SYTO 9 peak, by up to *c.* 7 times. Unsurprisingly, using the photobleaching half-lives of mB-SYTO 9 or mB-PI returned the lowest error in multi-spectra SVR, as the bound dye signals are a result of bacteria-dye interactions.

### Limitations and future improvements

As shown by the model validation results, the SVR models performed better in predicting the percentage of live bacteria in 10^8^ bacteria/mL samples compared to the 10^7^ bacteria/mL samples. The predictions for 10^8^ bacteria/mL samples struggle when there is a low proportion of live bacteria present, as shown by the test set sample containing 6.9% of live, which was not predicted within 2 standard errors of the 1:1 line. The model of 10^7^ bacteria/mL samples performs poorly and returns invalid predictions when the percentage of live bacteria is outside the measurable range of *c*. 7 to 73%. The SYTO 9 signal dominates the fluorescence emission when there is a high percentage of live bacteria, whereas the PI signal dominates when there is a high percentage of dead bacteria. Thus, making it more difficult to accurately obtain information from the dye signals at the extreme ends where there is either < 7% or > 73% of live bacteria present.

As the amount of dye used in this series of experiments was unchanged, the 10^7^ bacteria/mL samples contained significantly more unbound dye than the 10^8^ bacteria/mL samples. Thus, the signal to noise ratio is smaller in samples with lower bacterial concentration, due to the presence of a greater proportion of unbound dye. In an attempt to increase the signal to noise ratio, multiple spectra from one optrode measurement recorded from 10^7^ bacteria/mL samples were summed then used in analysis; however, this did not improve the prediction results. One way to experimentally overcome this limitation is to decrease the concentration of dyes used in samples that contain low bacterial concentrations. This requires prior knowledge of the approximate bacterial concentration of the sample, and further investigations would be necessary to find the optimal dye volumes for different concentrations. Another option is to remove unbound dye from the samples via washing; however, this requires extra sample processing and is difficult to complete without losing bacteria in the process [21]. Work is underway in our group to automate the dye staining and washing processes by using microfluidic platforms.

**Fig. 4:**
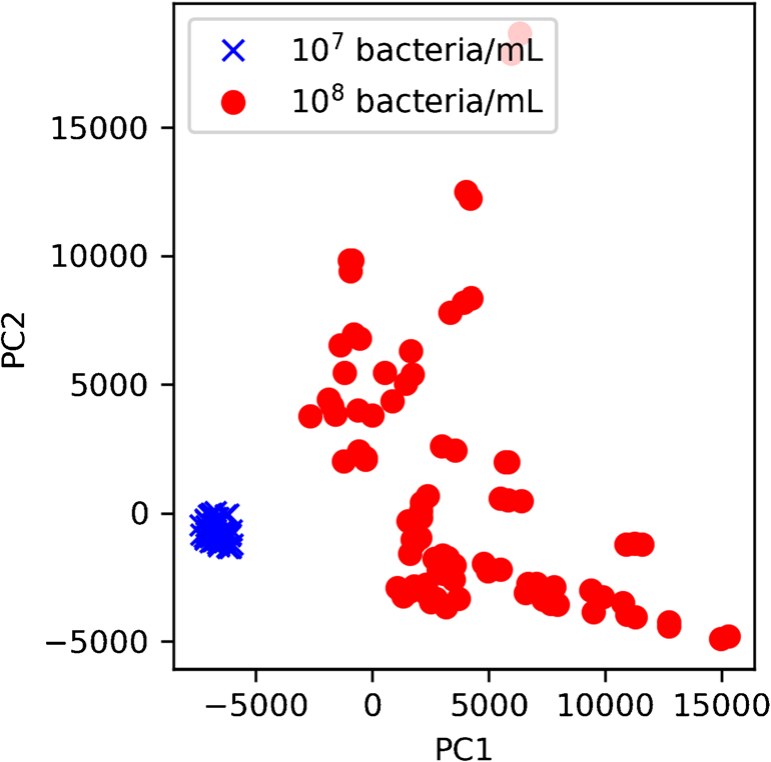
Principal component analysis scores plot of the first spectrum obtained from bacterial samples used to build the regression models. Although the samples contain varying live:dead bacterial cell ratios, there is a clear separation between samples that have a total concentration of 10^8^ and 10^7^ bacteria/mL

To further improve the accuracy for predicting the proportion of live bacteria present, the multi-spectra SVR analysis can be combined with principal components analysis (PCA). Already, we observed that the PCA scores of fluorescence spectra collected from blind samples can be used to inform which concentration group the samples belong to and accordingly, which SVR model to use for analysis. As shown in Fig. 4, the spectra obtained from 10^8^ and 10^7^ bacteria/mL samples can be easily separated using the first two components obtained from the PCA. Recent work also demonstrated the possibility to predict the concentration of live and dead bacteria in a bacterial mixture, using principal components regression [13]. By combining the information of bacterial concentration with the percentage of live bacteria present, it would be possible to obtain a more accurate and precise measurement of bacterial content in a sample.

In this study, we demonstrated the feasibility of using quantitative fluorescence spectra to monitor bacterial viability. We show this using spectral data obtained by the optrode, a convenient and cost-effective fibre-based spectroscopic device that accurately quantifies fluorescence signals in near real time. *E. coli* samples containing various proportions of live:dead cell concentrations were stained with SYTO 9 and PI, then subsequently measured using the optrode. Of the four analysis methods investigated, multi-spectra SVR obtained the most reliable results in predicting the percentage of live bacteria present. For 10^8^ bacteria/mL samples, the model was able to predict the percentage of live bacteria present down to *c.* 7% live. Predictions of the percentage of live bacteria in 10^7^ bacteria/mL were also achieved, though with a narrower detection range of *c.* > 7% and < 73% live. The main drawback from obtaining sensitive prediction at lower concentrations is the presence of unbound dyes, which can be reduced by decreasing the volume of dyes used or the use of an improved automated staining/washing process. This study demonstrates the value of an approach to improving the spectral processing method for monitoring the ratios of live:dead bacteria stained with SYTO 9 and PI. Further, we demonstrate that the multi-spectral SVR method and the optrode could be applied to monitor the effectiveness of a wide range of antimicrobial processes, including antibiotic treatment and food processing methods.
